# Comparative analysis of two radial hemostasis strategies following percutaneous coronary intervention: a prospective, single-center, real-world cohort study

**DOI:** 10.3389/fcvm.2026.1800752

**Published:** 2026-08-20

**Authors:** Pablo Juan-Salvadores, Samuel Pintos-Rodríguez, Cristina Herrera Álvarez, Julio Echarte Morales, Silvia González Suárez, María Luisa Núñez Seoane, Paula Pérez Rey, Rebeca Míguez Baquero, Víctor Alfonso Jiménez Díaz, Virginia Argibay Pytlik, Andrés Iñiguez Romo

**Affiliations:** 1 Cardiovascular Research Unit, Cardiology Department, Hospital Álvaro Cunqueiro, University Hospital of Vigo, Vigo, Spain; 2 Cardiovascular Research Group, Galicia Sur Health Research Institute (IIS Galicia Sur), SERGAS-UVIGO, Vigo, Spain; 3 Interventional Cardiology Unit, Department of Cardiology, Hospital Álvaro Cunqueiro, Área Sanitaria de Vigo, Vigo, Spain

**Keywords:** bleeding, complications, haemostasia, percutaneous coronary intervention, radial compression, radial hemostasis device

## Abstract

**Background:**

Radial access is currently the approach of choice for coronary interventional procedures. However, puncture and hemostasis are not exempt from access site complications. This study evaluated the efficacy and safety of two radial hemostasis devices used after percutaneous coronary intervention (PCI) in routine clinical practice.

**Methods:**

A prospective, single-center, real-world cohort study was carried out. Consecutive patients undergoing coronary angiography or PCI via radial access between February 2023 and October 2024 were included. Depending on the hemostasis technique used, the patients were assigned to TR-Closure band™ or a dedicated pad (VIRUNDA®). Access site complications were assessed at the time of device removal.

**Results:**

A total of 597 patients were included (TR-Closure band™ *n* = 282; VIRUNDA® *n* = 315). The median age was 71 years (63–78); 409 (68.5%) were male. Compression time was shorter with VIRUNDA® [median 113 [90–133] vs. 148 [125–180] minutes; *p* < 0.001]. VIRUNDA® was associated with lower bleeding rates [Bleeding Academic Research Consortium (BARC) score 1: 9.8% vs. 58.2%], less pain during compression (10.5% vs. 19.9%) and after compression (5.4% vs. 13.8%), and less paresis (2.5% vs. 8.5%) (all *p* ≤ 0.001). Hematoma was more frequent with TR-Closure band™ (27.7% vs. 19.4%; *p* = 0.009). The immediate radial artery occlusion rates were similar (9.2% vs. 4.6%; *p* = 0.143). The multivariate analysis identified the use of VIRUNDA® as an independent protective factor (RR 0.171; 95%CI 0.119–0.245; *p* < 0.001).

**Conclusions:**

Compared with TR-Closure band™, VIRUNDA® reduced bleeding, pain and paresis, and shortened compression time, without a significant increase in immediate radial occlusion. These findings support its use as a safe and efficient hemostatic alternative in routine practice.

## GRAPHICAL ABSTRACT

**Figure:**
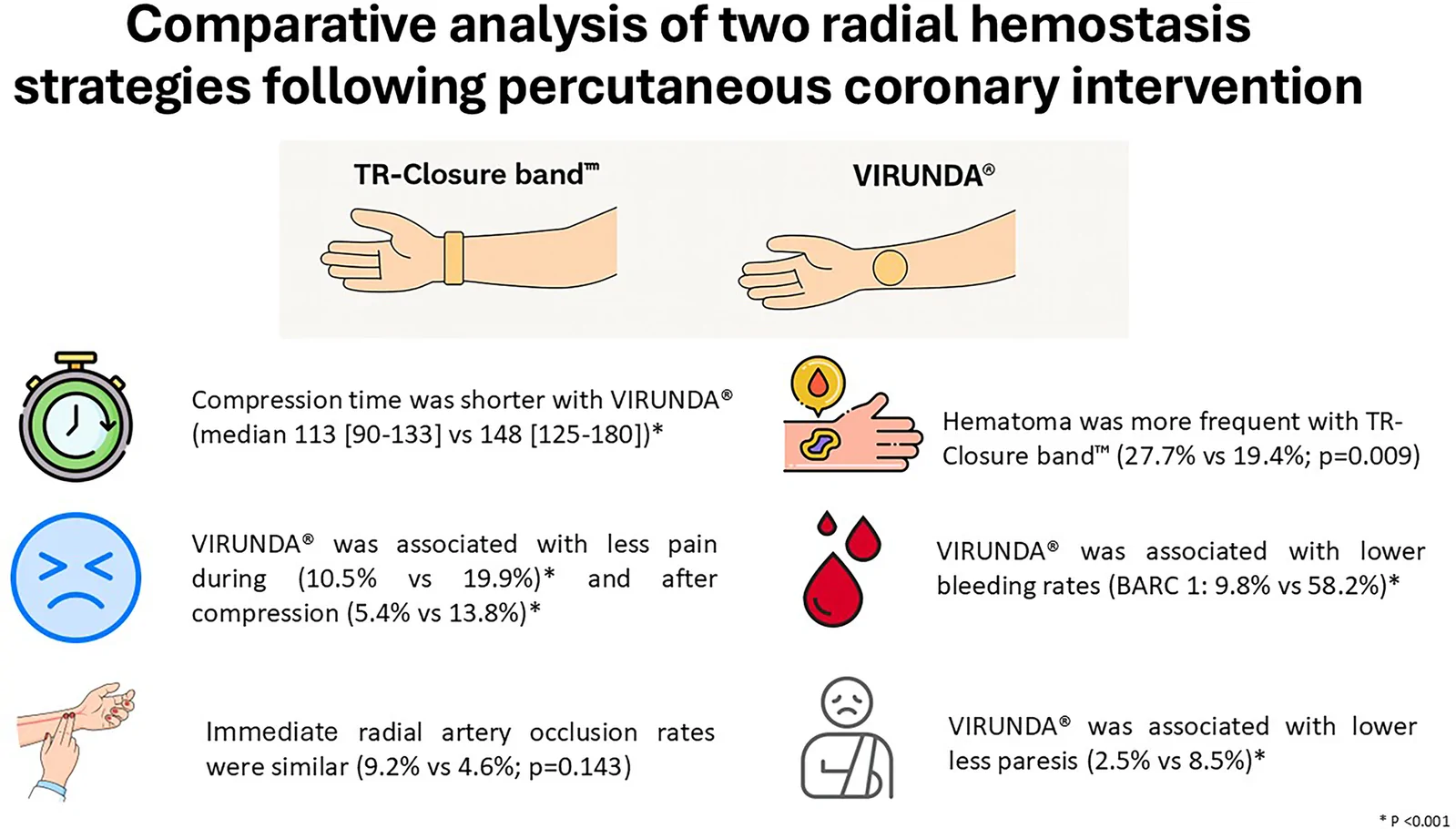

## Introduction

The radial artery has become consolidated as the vascular access route of choice in percutaneous coronary intervention (PCI), as it results in fewer vascular complications and is more comfortable for the patient compared with the femoral route (1–4). However, radial compression after removing the introducer remains a challenge in terms of efficacy, safety and comfort for the patient. In this regard, radial artery occlusion (RAO), spasm, hematoma, bleeding and pain may result, among other complications. RAO is the most frequent serious complication, with a reported incidence of 5%–12% in the immediate phase and a persistence rate of about 1%–5% over the long term (5–7). Radial spasm is observed in 4%–20% of the cases and is generally transient (5). Mild hematomas have been reported in 10%–20% of the cases, with moderate or severe hematomas in <3%, while the incidence of minor bleeding is about 8%–12% (5, 8). In addition, 20%–25% of the patients report pain or discomfort during compression (9, 10). All these complications can prolong hemostasis time, imply a need for hospital admission in procedures initially considered to be ambulatory, or delay discharge in hospitalized patients.

Many products have been marketed for radial artery hemostasis, such as pneumatic bands (TR Band®, Radistop®, TR-Closure band™) or patch devices (D-Stat®, Syvek Patch®) (11). However, studies on their efficacy and safety have yielded heterogeneous results (9, 12–14). Technological developments have led to the design of new hemostatic devices that could offer clinical and economic advantages.

The present study was conducted to compare immediate access-site outcomes associated with two radial hemostasis strategies (VIRUNDA®) vs. TR-Closure band™, in patients subjected to coronary arteriography or PCI via the radial access.

## Methodology

**Figure 1:**
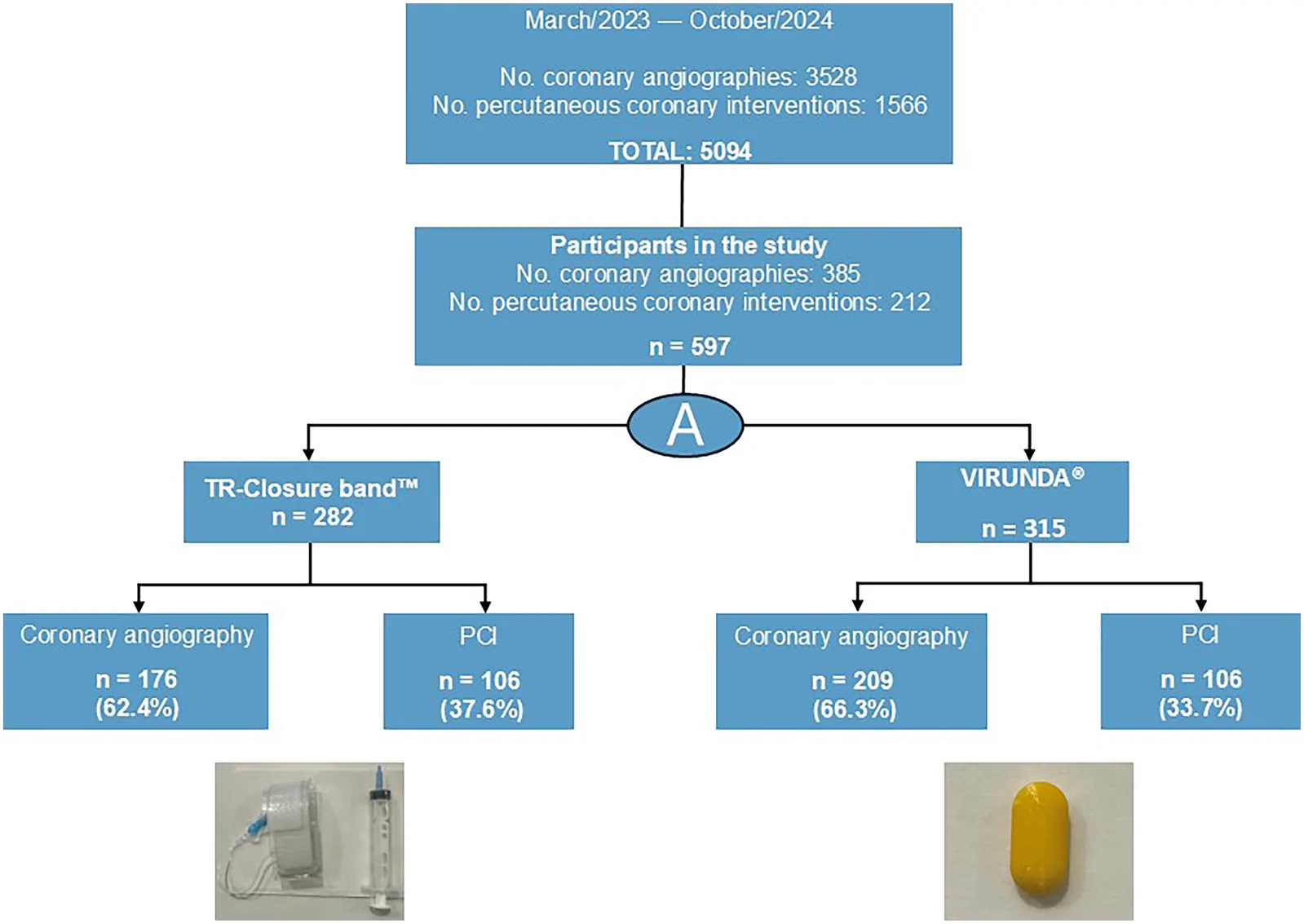
Study population flow chart.

A pragmatic, prospective, single-center cohort study conducted in routine clinical practice was carried out in the Hemodynamics and Interventional Cardiology Unit of a tertiary hospital. The inclusion period extended from February 2023 to October 2024, because only nurses with proven experience in both radial hemostasis techniques were allowed to participate. Rotating or trainee staff were excluded to ensure technical consistency and reproducibility of both procedures. During this period, we included all patients with an indication of cardiac catheterization (coronary arteriography and/or PCI) via the radial route who agreed to participate in the study (Figure 1).

The patients were divided into two groups according to the type of hemostatic compression device used after the procedure: TR-Closure band™ group or VIRUNDA® group. The initial hemostasis strategy was selected according to a calendar-based alternating-day (one day TR-Closure band™ and another day VIRUNDA®) implementation schedule used in routine practice. This schedule was not a randomization procedure, and therefore calendar-related, procedural, operator-related, staff-related, and other unmeasured confounding cannot be excluded. The following inclusion and exclusion criteria were applied:

Inclusion criteria: Men and women ≥18 years of age. Indication of coronary arteriography or PCI. Patients able to understand the study, who agreed to participate and signed the informed consent document.

Exclusion criteria: Patients operated upon via the femoral, brachial or ulnar route. Switching from the radial access to the femoral, brachial or ulnar route during the percutaneous procedure. Allergy to the pads commonly used in hemostasis practice. A previous diagnosis of coagulopathy. Patients with a platelet count of <150 × 10^9^/L. Pregnant or breastfeeding women.

### Compression protocols

1. TR-Closure band™: (a) After cleaning the puncture site, the pad is placed on the radial puncture orifice, with the insufflation extension towards the palm of the hand. (b) The syringe specific to the device is filled with 18 mL of air. (c) The introducer is removed after insufflating the pneumatic balloon to 13 mL (in the event of bleeding, increase by 2 mL until bleeding stops). (d) After 30 min, 7 mL of air is withdrawn and compression is maintained until 2 h are completed. (e) At the end of mechanical compression, two slightly crossed adhesive strips are placed (avoiding circular bandaging of the wrist) over a piece of dressing folded four times and previously placed over the arterial puncture site. (f) The dressing is to be left in place for 24 h. In the case of skin sensitive to all types of dressings, the radial zone should be previously sprayed with Nobecutan®.
2. VIRUNDA®: (a) After cleaning the puncture site, it is sprayed with Nobecutan®. (b) A cellulose dressing is placed. (c) VIRUNDA® is positioned on top of the dressing for arterial compression; the rounded portion of the VIRUNDA® device is to be in contact with the dressing. (d) VIRUNDA® is affixed with three elastic adhesive strips: 2 in cross-form and 1 transversal. (e) The device is removed after two hours of compression, and the same dressing as that used following the TR-Closure band™ is then applied and likewise kept in place for 24 h. In the case of skin sensitive to all types of dressings, the radial zone should be previously sprayed with Nobecutan®. The full protocol with images is shown in supplementary material I.

### Sample size

To achieve a statistical power of 80% in rejecting the null hypothesis H_0_, a non-inferiority limit was established with a normal asymptotic test for unilateral proportions for two independent samples, taking into account a significance level of 5% and assuming that the proportion in the TR-Closure band™ group is 30% vs. 32% in the VIRUNDA® group, and with a proportion of experimental units in both groups of 50%, with a non-inferiority limit of 11%. A total of 287 units would thus be needed in the TR-Closure band™ group and 287 units in the VIRUNDA® group, representing a total of 574 units in the study. However, because the present analysis was based on a non-randomized pragmatic cohort, no formal non-inferiority inference was performed, and all comparisons should be interpreted as observational associations.

### Primary and secondary endpoints

The primary endpoint of the study was the incidence of complications related to the percutaneous intervention, defined as the presence of one or more of the following adverse events: bleeding, hematoma, paresis, local pain and immediate occlusion of the radial artery.

Bleeding: Defined as continuous blood loss at the radial artery puncture site, assessed by the Bleeding Academic Research Consortium (BARC) scale (15). Hematoma: Defined as visible swelling at the puncture site, with loss of normal skin texture, and classified into four grades according to the EASY scale (16): Grade I: superficial local hematoma measuring <5 cm from the puncture site. Grade II: hematoma with moderate muscle infiltration measuring <10 cm from the puncture site. Grade III: hematoma of the forearm with muscle infiltration reaching the elbow. Grade IV: compartment syndrome. Paresis: Defined as loss of muscle strength in the arm, with limited range of motion. This variable was recorded qualitatively as either present or absent. Pain: Evaluated using the Wong-Baker Faces visual analogue scale and according to the World Health Organization (WHO) definition of pain as an unpleasant sensory and emotional experience associated with tissue damage (17). Radial artery occlusion (RAO): Defined as the absence of a palpable radial pulse, confirmed by a negative reverse Barbeau test, negative plethysmography, absence of Doppler signal and/or direct visualization of the obstruction using ultrasound. RAO was classified into types A to D according to intensity. All clinical evaluations, outcome assessments and procedural measurements were performed using the same standardized protocol in both groups.

The following demographic and clinical variables were recorded for each patient: age, sex, weight, cardiovascular risk factors (arterial hypertension, diabetes mellitus, smoking), past cardiovascular events, and regular treatment with antiplatelet drugs and anticoagulants before the intervention.

The following data were recorded in relation to the cardiac catheterization procedure involved: type of procedure (diagnostic coronary angiography, PCI), radial route used (right or left), caliber of the catheter or introducer used, number of affected vessels, heparin dosing during the procedure, and antiplatelet drug loading dose, where applicable. In addition, we recorded parameters related to the post-catheterization arterial compression procedure: compression start and end time, and the need for early removal of the hemostatic device or its replacement by another alternative method due to active bleeding.

### Statistical analysis

A descriptive analysis of the data was performed, reporting qualitative variables as absolute frequencies and percentages, and quantitative variables as the mean and standard deviation (SD) or percentiles in the absence of a normal distribution. The Chi-squared test was used to compare two qualitative variables.

Significant differences between the two groups were evaluated by means of a bivariate analysis, comparing qualitative variables, analyzing the normality of distribution of the data in each of the groups, and applying parametric [Student t-test, analysis of variance (ANOVA)] or nonparametric tests (Mann–Whitney U-test, Kruskal–Wallis test), as applicable. Those variables that showed an association with disease prevalence or severity in the univariate analysis were subsequently entered as independent variables in the multivariate regression analysis. A multivariate logistic regression model was used to assess the contribution of the risk combination. The analyzed risk factors were expressed as the odds ratios (OR), with the corresponding 95% confidence interval (95% CI). Missing data were minimal and were handled using a complete-case approach. No imputation methods were applied. In all cases of hypothesis testing, the *α* significance level accepted was 0.05. The SPSS version 19 statistical package for MS Windows was used throughout.

### Ethical and legal issues

The study was carried out in cordance with the principles of the Declaration of Helsinki, the Oviedo Convention, and the Good Clinical Practice guidelines. Current legislation regarding the protection of personal data was strictly observed in all cases. The study protocol was previously evaluated and approved by the Clinical Research Ethics Committee of Pontevedra-Vigo-Ourense (reference 2022/485). All subjects gave written informed consent prior to inclusion in the study.

## Results

The study involved 597 patients subjected to radial catheterization, of which 282 (47.2%) underwent compression with TR-Closure band™ and 315 (52.8%) with VIRUNDA®.

### Clinical characteristics

The baseline demographic and clinical parameters of the patients according to the compression device employed after catheterization are shown in Table 1. The median age of the patients was 71 years (range 63–78), and 409 (68.5%) were male. Both groups were comparable, with no statistically significant differences in their baseline characteristics.

**Table 1:** Baseline clinical parameters according to the type of radial artery compression used after catheterization.

Variables	Total (*n* = 597)	TR-Closure band™ (*n* = 282)	VIRUNDA® (*n* = 315)	*P*-value
Age, years	71 (63–78)	71 (63–78)	72 (62–78)	0.910
Male sex	409 (68.5)	193 (68.4)	216 (68.6)	0.972
BMI, kg/m^2^	28.3 ± 4.9	28.1 ± 4.5	28.6 ± 5.3	0.232
Diabetes mellitus	170 (28.4)	81 (28.8)	89 (28.2)	0.877
Arterial hypertension	398 (66.7)	195 (69.2)	203 (64.4)	0.223
Dyslipidemia	398 (66.7)	193 (68.4)	205 (65.1)	0.385
Smoking				
Active smoker	112 (18.7)	44 (15.6)	68 (21.6)	0.128
Ex-smoker	177 (29.7)	91 (32.3)	86 (27.3)	
Atrial fibrillation	101 (16.9)	52 (18.4)	49 (15.6)	0.348
CKD	43 (7.2)	17 (6.0)	26 (8.3)	0.294
Peripheral artery disease	70 (11.7)	32 (11.4)	38 (12.1)	0.786
Previous AMI	103 (17.3)	45 (16.0)	58 (18.4)	0.428
Previous PCI	136 (22.8)	63 (22.3)	73 (23.2)	0.808
Previous CVA	35 (5.9)	17 (6.0)	18 (5.7)	0.870
Previous surgical revascularization	36 (6.0)	18 (6.4)	18 (5.7)	0.732

Data reported as *n* (%) or mean ± SD or median [IQR].

ASA, acetylsalicylic acid; SD, standard deviation; CKD, chronic kidney disease; AMI, acute myocardial infarction; PCI, percutaneous coronary intervention; BMI, body mass index; IQR, interquartile range; TR, transradial.

The characteristics of the interventions are summarized in Table 2. The catheterization indications were comparable between the groups (*p* = 0.196), with the following distribution: stable angina (66.7% for TR-Closure band™ vs. 58.4% for VIRUNDA®), NSTEMI (31.6% vs. 39.0%) and STEMI (1.8% vs. 2.5%). The peri-procedure administration of anticoagulants proved similar (*p* = 0.918), as was the use of P2Y12 platelet inhibitors (55.2% vs. 55.1%; *p* = 0.881). Acetylsalicylic acid was slightly more often used in the VIRUNDA® group (80.3% vs. 72.7%; *p* = 0.028).

**Table 2:** Characteristics of the procedure according to the type of radial artery compression used after catheterization.

Variables	Total (*n* = 597)	TR-Closure band™ (*n* = 282)	VIRUNDA® (*n* = 315)	*P*-value
Indication of catheterization				
Stable angina	372 (62.3)	188 (66.7)	184 (58.4)	0.196
NSTEMI	211 (35.5)	89 (31.6)	123 (39.0)	
STEMI	13 (2.2)	5 (1.8)	8 (2.5)	
Peri-procedure medication				
Anticoagulant				
No	516 (86.4)	245 (86.9)	271 (86.0)	0.918
DOAC	49 (8.2)	22 (7.8)	27 (8.6)	
Vitamin K antagonist	31 (5.2)	14 (5.0)	17 (5.4)	
P2Y12 inhibitors	331 (55.4)	157 (55.2)	174 (55.1)	0.881
ASA	458 (76.7)	205 (72.7)	253 (80.3)	0.028
Loading dose	17 (2.9)	10 (3.6)	7 (2.23)	0.335
Procedure type				
Diagnostic coronary angiography	385 (60.5)	176 (62.4)	209 (63.3)	0.229
PCI	212 (35.5)	106 (37.6)	106 (33.7)	
Heparin during the procedure	580 (97.2)	272 (96.5)	308 (97.8)	0.218
Heparin dose, IU/mL	5,000 (4,000–8,000)	5,000 (4,000–8,000)	5,000 (4,000–8,000)	0.737
Duration of the procedure, minutes	29 (16–48)	29 (16–48)	29 (16–47)	0.786
Number of diseased vessels				
0	206 (34.5)	102 (36.2)	104 (33.0)	0.413
1	204 (34.2)	96 (34.0)	108 (34.3)	
2	103 (17.3)	51 (18.1)	52 (16.5)	
3	82 (13.7)	32 (11.3)	50 (15.9)	
Access				
Right radial	543 (91.0)	251 (89.0)	292 (92.7)	0.248
Left radial	54 (9.0)	31 (11.0)	23 (7.3)	
Number of punctures to achieve vascular access				
1	564 (94.6)	263 (93.6)	301 (95.6)	0.289
2	33 (5.4)	19 (6.4)	14 (4.4)	
Catheter caliber				
5 Fr	268 (45.0)	126 (44.7)	142 (45.4)	0.565
6 Fr	325 (54.6)	155 (55.0)	170 (54.3)	
Hemoglobin during the procedure, g/dL*	13.8 (12.8–14.9)	13.8 (12.6–14.8)	13.9 (12.9–14.9)	0.384
Duration of compression, minutes	128 (102–156)	148 (125–180)	113 (90–133)	<0.001

Data reported as *n* (%) or mean ± SD or median [IQR].

* Hemoglobin during the procedure was available in 516 patients.

ASA, acetylsalicylic acid; SD, standard deviation; Fr, French; NSTEMI, non-ST elevation myocardial infarction; STEMI, ST elevation myocardial infarction; PCI, percutaneous coronary intervention; IQR, interquartile range; TR, transradial.

There were no differences in the type of procedure performed (PCI 37.6% vs. 33.7%; *p* = 0.229), heparin dose administered [5,000 (4,000–8,000) IU in both groups; *p* = 0.737], duration of the procedure [29 [16–48] vs. 29 [16–47] min; *p* = 0.786] or caliber of the catheters used (5 Fr: 44.7% vs. 45.4%; 6 Fr: 55.0% vs. 54.3%; *p* = 0.565). The duration of compression was significantly shorter in the VIRUNDA® group [113 [90–133] vs. 148 [125–180] min; *p* < 0.001].

### Clinical events

**Figure 2:**
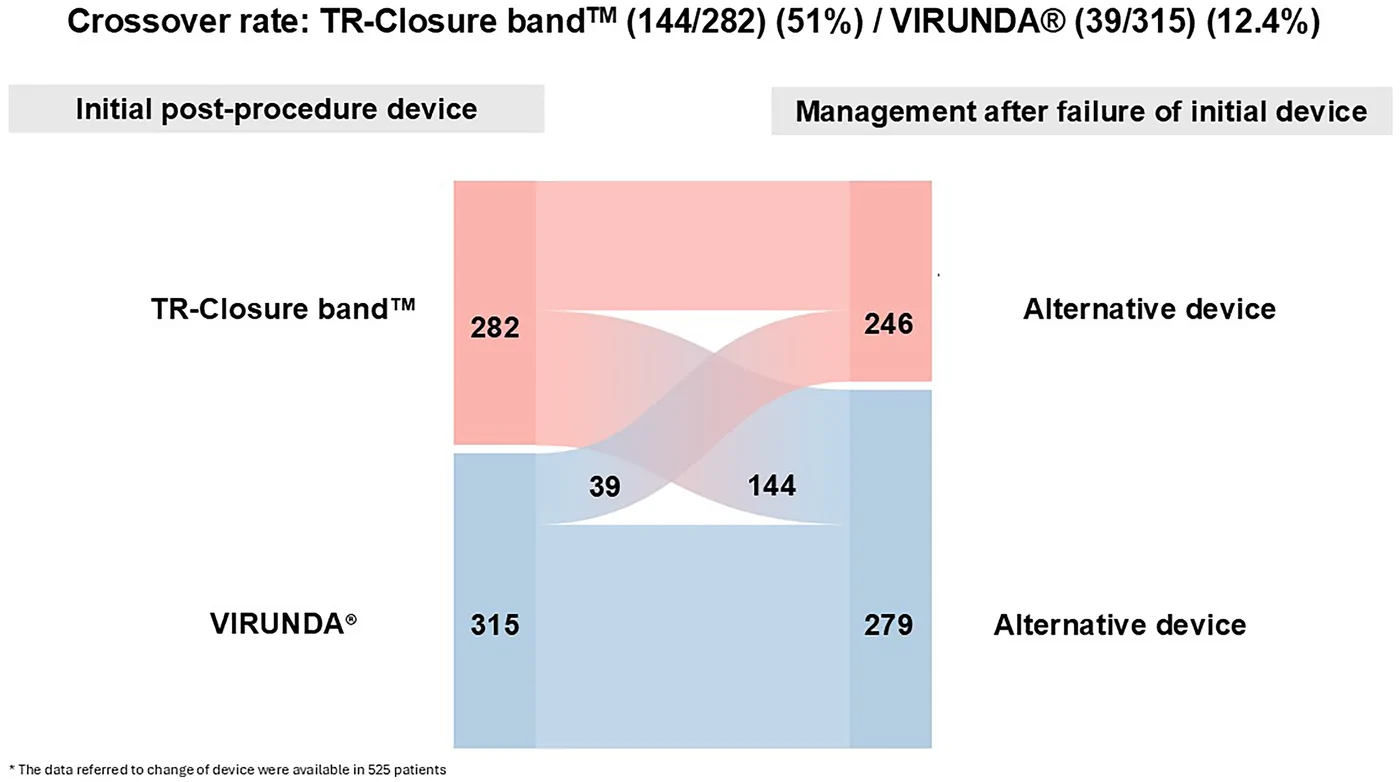
Changes in compression device.

Bleeding (BARC ≥1) was more frequent with TR-Closure band™ than with VIRUNDA® (58.9% vs. 10.2%; *p* < 0.001). The incidence of hematoma (27.7% vs. 19.4%; *p* = 0.009), pain during compression (19.9% vs. 10.5%; *p* < 0.001) or pain after compression (13.8% vs. 5.4%; *p* < 0.001) was also higher with TR-Closure band™. Paresis was recorded in 8.5% of the patients with TR-Closure band™ vs. in 2.5% of those with VIRUNDA® (*p* = 0.001). There were no significant differences in the incidence of RAO (4.6% vs. 9.2%; *p* = 0.143). The need for vascular intervention was low and was observed only in the TR-Closure band™ group (1.8% vs. 0%; *p* = 0.030) (Table 3). In the event of hemostasis failure with the assigned compression device, rescue manual compression was performed as shown in Figure 2.

**Table 3:** Results of compression according to the device used.

Variables	Total (*n* = 597)	TR-Closure band™ (*n* = 282)	VIRUNDA® (*n* = 315)	*p*-value
Bleeding	198 (33.2)	166 (58.8)	32 (10.2)	<0.001
No bleeding	399 (66.8)	116 (41.2)	283 (89.8)	<0.001
Type 1 bleeding	197 (99.5)	165 (99.4)	32 (100)	<0.001
Type 2 bleeding	1 (0.5)	1 (0.4)	0 (0.0)	0.123
Hematoma	139 (23.2)	78 (13.1)	61 (10.2)	0.009
Grade I	122 (21.2)	70 (26.2)	52 (16.9)	0.022
Grade II	10 (1.7)	5 (1.9)	5 (1.6)	
Radial artery occlusion	42 (7.0)	13 (4.6)	29 (9.2)	0.143
Pain during compression	89 (14.9)	56 (19.9)	33 (10.5)	<0.001
Post-compression pain	56 (9.4)	39 (13.8)	17 (5.4)	<0.001
Paresis	32 (5.4)	24.2 (8.5)	8 (2.5)	0.001
Intervention	5 (0.8)	5 (1.8)	0 (0.0)	0.030

Data are presented as *n* (%).

### Predictors of radial complications after catheterization

In the univariate analysis, only the type of hemostatic device employed (VIRUNDA® vs. TR-Closure band™) was significantly associated with a lesser risk of radial artery complications (RR 0.178; 95% CI, 0.125–0.252; *p* < 0.001). In the multivariate model (Table 4), the independent predictors of radial artery complications were patient age (RR 1.026 per year: 95% CI, 1.010–1.044; *p* = 0.003) and the type of device employed, with a consistent protective effect being observed for VIRUNDA®, (RR 0.171; 95% CI, 0.119–0.245; *p* < 0.001).

**Table 4:** Predictors of radial complications after catheterization.

Variables	Univariate		Multivariate	
	Odds Ratios (95%CI)	*p*-value	Odds Ratios (95%CI)	*p*-value
Age, years	1.000 (0.997–1.001)	0.586	1.026 (1.102–1.044)	0.003
Male sex	1.177 (0.833–1.664)	0.354	1.173 (0.783–1.756)	0.439
BMI, kg/m^2^	0.976 (0.944–1.010)	0.161	0.985 (0.949–1.023)	0.438
Diabetes mellitus	0.967 (0.677–1.381)	0.855	0.926 (0.749–1.146)	0.481
Atrial fibrillation	0.990 (0.645–1.519)	0.962	0.833 (0.434–1.531)	0.557
CKD	1.26 (0.765–2.079)	0.362	0.892 (0.438–1.816)	0.753
Peripheral artery disease	1.261 (0.765–2.079)	0.362	1.442 (0.816–2.548)	0.207
Diagnostic catheterization versus PCI	1.183 (0.843–1.662)	0.330	1.000 (0.975–1.026)	0.999
Anticoagulant	0.915 (0.785–1.066)	0.254	0.893 (0.718–1.111)	0.310
ASA	0.940 (0.643–1.374)	0.750	1.083 (0.655–1.792)	0.756
Loading dose	2.041 (0.745–5.592)	0.165	1.763 (0.571–5.446)	0.324
Heparin dose >5,000 IU	1.098 (0.778–1.545)	0.595	1.091 (0.708–1.684)	0.692
VIRUNDA® vs TR-Closure band™	0.178 (0.125–0.252)	<0.001	0.171 (0.119–0.245)	<0.001

ASA, acetylsalicylic acid; CKD, chronic kidney disease; PCI, percutaneous coronary intervention; BMI, body mass index.

## Discussion

The main findings of the present study can be summarized as follows: (1) Hemostasis using a specifically designed device (VIRUNDA®) was associated with less mild bleeding, pain and paresis, and fewer hematomas. (2) VIRUNDA® shortened the hemostatic compression time compared with TR-Closure band™. (3) There were no significant differences in immediate RAO between the two devices.

The profile of the study cohort reflects usual clinical practice and is consistent with the published series (9, 18). The heterogeneity of the radial artery hemostatic devices and methods used in the different studies makes it difficult to extrapolate the results obtained. In our cohort, we consider the shortening of radial artery compression time by over 35 min with VIRUNDA® vs. TR-Closure band™ to be important. In other studies on hemostasis, the compression times have ranged between 80 and 180 min (6, 9, 14, 19), with shorter times of 80 min on average when compression is performed manually (20)—this being consistent with the times obtained in our series using VIRUNDA®.

In our study, the immediate RAO rate was low, with no significant differences between the two groups, and with incidences in line with the data reported in the literature (5%–12%) (8, 13, 19). Although the rate was numerically higher in the VIRUNDA® group, this difference did not reach statistical significance and should be interpreted cautiously in the context of the observational design and the immediate timing of assessment. The variations found in the published series are largely conditioned by the timing of the evaluation, the type of hemostasis, and the duration and intensity of compression (5). In this regard, non-occlusive hemostatic strategies and a shortening of the compression time have been associated with a lesser incidence of RAO (5, 8, 9, 12, 20, 21). Possible explanations for the numerical imbalance observed in our cohort include differences in local pressure distribution, anatomical fit, individual radial artery characteristics, protocol adherence, or unmeasured procedural factors; however, these mechanisms were not directly assessed. Importantly, radial patency was evaluated only at device removal, without systematic Doppler ultrasound follow-up. Therefore, we cannot determine whether these early findings persisted or recanalized over time, and no conclusions can be drawn regarding long-term radial artery patency.

In our cohort, the use of VIRUNDA® was associated with a lesser incidence of bleeding (which proved mild in all cases) upon suspending compression than TR-Closure band™. The relatively high incidence of BARC 1 bleeding observed in the TR-Closure band™ group can be partly explained by the timing and definition used in this study. Bleeding was assessed immediately after device removal, whereas most previous studies evaluate at 24 h, when minor oozing has usually resolved. Furthermore, our definition intentionally included any visible blood loss, even if clinically insignificant. This sensitive approach may overestimate minor events but allows a more accurate comparison of device-related performance. Similar results have been reported in another study comparing a pneumatic band with other alternative devices (13). However, other studies have not found significant differences in bleeding between devices (9, 12, 20), which suggests that the intensity and duration of compression may play a relevant role together with the protocolization of hemostatic compression in keeping the bleeding rates low (5).

Hematomas are frequent complications after radial artery access and may be related to excessive pressure or suboptimal placement and insufflation of the device (22). In relation to proximal radial artery access, the MEMORY trial compared manual compression against mechanical compression and recorded practically identical hematoma rates after 24 h (17% vs. 18%; *p* = 0.749) (9). In contrast, in distal radial artery access, Roghani-Dehkordi et al. recorded more hematomas with manual compression than with TR-Band® (13.5% vs. 5.4%; *p* = 0.431) (14). The discrepancies with respect to our own cohort may be due to the timing of evaluation vs. other studies that perform evaluation after 24 h or even later, when many grade I hematomas have already subsided. In our case, evaluation was made after removing the device, and this may have contributed to increase the observed incidence (9, 14).

Post-procedure pain is not uncommon after radial artery compression, and is related to the intensity and duration of compression; adjusting the pressure and time therefore may favor patient comfort (5). A randomized study found moderate to intense pain to be more frequent when using a pneumatic band than with an alternative device, in concordance with our own observations (22). However, few studies have assessed pain with a validated scale, which makes it difficult to establish comparisons (13, 18).

Neurological dysfunction of the hand after radial artery access is infrequent and is usually transient (14). Dehkordi et al. reported more frequent paresis and/or numbness with manual compression than when using a pneumatic band device, which underscores the role of compression pressure and duration (14). Longer compression or occlusion increases the risk of local symptoms, hence the recommendation to use protocols involving minimum pressure and stepwise withdrawal (5).

### Limitations

Our study has several limitations. First, its single-center design limits the external validity of the findings and may introduce center-specific biases. Although the study reflects routine clinical practice and included a relatively large cohort, these characteristics do not eliminate the possibility of residual confounding. In addition, the calendar-based alternating-day implementation schedule was not a randomization procedure and may have been influenced by unmeasured temporal, procedural, operator-related, or staff-related factors. Third, the frequent need for a change in the initial compression strategy in the TR-Closure band™ group limits the interpretation of the results as a direct device-to-device comparison, since subsequent outcomes may reflect both the initial strategy and the rescue procedure. Compression duration was not adjusted according to heparin dosage or sheath size, as the study sought to reproduce routine practice at our institution. This lack of standardization may have influenced bleeding rates and should be considered when interpreting the results. Fourth, radial artery occlusion was assessed only immediately after device removal. No systematic ultrasound follow-up was performed at 24 h or later; therefore, persistent, delayed, or subclinical RAO could not be assessed. Although early RAO may resolve over time in some patients, the absence of serial ultrasound assessment prevents estimation of its late incidence or trajectory in either group. The observed difference in immediate RAO between groups should consequently be interpreted cautiously and does not allow conclusions regarding long-term radial artery patency. Immediate post-compression evaluation may overestimate minor bleeding and small hematomas compared with assessments performed at 24 h, potentially inflating early complication rates in both groups. Finally, the composite endpoint included minor bleeding and patient-reported symptoms, which may have different clinical relevance and should therefore be interpreted separately. Multicenter randomized studies with standardized patent-hemostasis protocols, prespecified rescue procedures, and serial ultrasound assessment of radial patency are needed to confirm these findings across diverse clinical settings.

## Conclusions

In our experience, and in the context of the studied patient cohort, hemostasis using a specifically dedicated manual compression device (VIRUNDA®) was associated with less bleeding, pain and paresis, and fewer hematomas than the TR-Closure band™ device. In addition, it shortened the hemostatic compression time without increasing the incidence of immediate RAO. Overall, the findings obtained support the use of an efficient and safe solution for hemostasis after percutaneous radial access.

## Data Availability

The data used to support the findings of this study are available from the corresponding author upon reasonable request.
