# Supplementary material for: Comparative analysis of two radial hemostasis strategies following percutaneous coronary intervention: a prospective, single-center, real-world cohort study
Source: Front Cardiovasc Med. 2026 Aug 20;13:1800752. doi: 10.3389/fcvm.2026.1800752 (PMC13539125; doi:10.3389/fcvm.2026.1800752)
Supplement: Supplementary file 1 [file Datasheet1.docx]

**RADIAL ARTERY HEMOSTASIS PROTOCOL WITH THE VIRUNDA® DEVICE**

Following radial artery access catheterization, hemostasis of the puncture zone is performed as follows:

1. Clean the skin in the puncture zone around the introducer using a moist piece of dressing, and dry with a pad or dressing.
2. Spray Nobecutan® onto the shaved zone (minimum distance 20 cm) and allow to dry for 30 seconds. This prevents the zone from remaining moist due to perspiration, and protects the skin through subsequent contact with the dressing.

3. Place a pre-cut piece of cellulose on the puncture site and then position VIRUNDA® over it with the flat part facing upwards; this surface will help to affix it and apply pressure to the radial artery (0.3 cm below the puncture), while removing the introducer at the same time.

4. Affix VIRUNDA® using three strips of elastic adhesive bandage such as Tensoplast® (5 cm wide), placing 2 strips obliquely and 1 transversely; spread the ends of the elastic strips and affix VIRUNDA® with the thumb at the central point of the strips, preventing its displacement from the radial pressure zone.

5. Remove the bandage and VIRUNDA® device, maintaining slight radial compression with the index and middle finger close to the bandage (at the lateral edge of the bandage towards the forearm, NOT the lateral edge close to the hand); this will avoid sudden return of blood flow in the artery on suspending the compression applied by VIRUNDA® with the 3 elastic strips, which could result in bleeding through loss of the hemostatic plug produced during compression.
6. Place a piece of sterile dressing folded 4 times, and affix it at the puncture site with 3 strips like those mentioned above (point 4), but using flexible and porous tape (e.g., Mefix).
